# Creating action plans in a serious video game increases and maintains child fruit-vegetable intake: a randomized controlled trial

**DOI:** 10.1186/s12966-015-0199-z

**Published:** 2015-03-18

**Authors:** Debbe Thompson, Riddhi Bhatt, Isabel Vazquez, Karen W Cullen, Janice Baranowski, Tom Baranowski, Yan Liu

**Affiliations:** USDA/ARS Children’s Nutrition Research Center, Baylor College of Medicine, 1100 Bates Street, Houston, TX USA; Department of Family and Community Medicine, Baylor College of Medicine, 3701 Kirby Drive, Houston, TX USA

**Keywords:** Videogame, Fruit, Vegetables, Children, Implementation intentions, Maintenance

## Abstract

**Background:**

Child fruit and vegetable intake is below recommended levels, increasing risk for chronic disease. Interventions to influence fruit and vegetable intake among youth have had mixed effects. Innovative, theory-driven interventions are needed. Goal setting, enhanced by implementation intentions (i.e., plans tightly connected to a behavioral goal), may offer a solution. Action plans state “how” a goal will be achieved, while coping plans identify a potential barrier and corresponding solution. The research reported here evaluated the short- and long-term effects of goal setting enhanced with implementation intentions on child fruit and vegetable intake in a 10-episode, theoretically-grounded serious videogame promoting fruit and vegetables. This is one of the first studies to test the efficacy of implementation intentions on the dietary intake of healthy children.

**Methods:**

A four-group randomized design with three data collection periods (baseline, immediate post-intervention, 3 months post-intervention) was employed. Groups varied on whether children created an implementation intention (none, action, coping, both) as part of goal setting. Participants were 4th and 5th grade children (~9-11 years old) and one parent. An a priori power analysis indicated this would provide >80% power to detect a small effect (Cohen’s d = 0.17). Children played a 10-episode online videogame; parents received 10 electronic newsletters and access to a parent-only website. The primary outcome was child fruit and vegetable intake, assessed via three, dietitian-assisted telephone recalls at each data collection period. The primary analysis was conducted using a repeated measures analysis of covariance with a mixed model procedure. Secondary analyses examined intervention effects on fruit and vegetables separately.

**Results:**

Four hundred parent/child dyads were recruited. A significant group-by-time interaction for fruit and vegetable intake (p < 0.001) was found in only the Action group, which had significant increases in fruit and vegetable intake at post 1 (p < 0.0001) and post 2 (p < 0.0001). No other significant interactions were observed; however, there were significant time effects for fruit (p < 0.0001).

**Conclusions:**

Action intentions may be an important component of successful interventions to increase and maintain fruit and vegetable intake in pre-adolescent children. Videogames promoting healthy diets offer an effective vehicle for delivering behavior change interventions to children.

**Trial registration:**

ClinicalTrials.gov NCT01004094.

## Background

Fruit and vegetable (FV) intake is part of a healthy lifestyle [1] and reduces risk of developing chronic diseases, such as cardiovascular disease, hypertension, and certain cancers [2,3]. Due to their low energy density, FV intake may also decrease total calorie intake [4], thus reducing risk of obesity in the adult years [5]. FV intake behaviors are established early in life [6], and tend to track into adulthood [7-9], thus increasing the importance of establishing these behaviors during childhood.

National recommendations for pre-adolescent children encourage consuming 7–11 servings of FV per day[10]. Using MyPyramid as a guide, this equates to daily minimums of 1.5 cup equivalents of fruit (F) and 2.0 – 2.5 of vegetables (V) [11]. However, few children met even the minimum recommendation [11]. Interventions to increase FV intake among children have had mixed effects [12,13]; thus, new and effective intervention approaches are needed.

Theory provides a framework for developing interventions focused on factors that influence a specific behaviour, such as FV intake [14]. Goal setting, a theory-driven technique [15], is a promising dietary behaviour change procedure [16,17]. Although goal setting is often included in dietary behaviour change interventions for children [18-24], few studies have tested the effect of goal setting on dietary intake or investigated ways to enhance its effectiveness with children [25-28]. Because child preferences for FV vary [29], interventions to increase child FV intake should be responsive to this by enabling children to meet their goals by choosing preferred FV.

Implementation intentions are detailed plans tightly connected to a specific goal [30,31]. Expanding goal setting to include implementation intentions may enhance goal attainment [30-32] and thus, intervention effectiveness. There are two types of implementation intentions: action plans and coping plans. Action plans are highly specified plans that describe how (i.e., what, when, where) the goal will be attained (i.e., an action plan). Youth encounter barriers when attempting to eat FV [33], and problem solving is a common method for developing a plan to overcome them [34,35]. Coping plans, a type of problem solving, are designed to help children overcome common barriers or problems that may interfere with FV consumption [21,36]. Coping plans take an “if/then” approach, and guide the child through a process that identifies a specific barrier that may occur to hinder goal attainment and a course of action that will be taken if the barrier is encountered [30,37]. Implementation intentions have been effective at enhancing a variety of goal-directed behaviors, particularly among adults [31]. However, there is little evidence of effectiveness with healthy children, particularly in regards to diet [31,38,39]. More research is needed to investigate this promising technique.

Interventions to increase FV intake among children are often delivered in-person [12]; however, alternative approaches may be needed to effectively promote increased FV intake in large numbers of children. Videogames may offer an alternative. Children enjoy videogames; play a wide variety of videogame genres; play them often; and live in homes with high speed internet access [40]. Although excessive time spent in entertainment-oriented videogames has been associated with negative health effects [41], serious videogames, an emerging genre, offer a promising intervention approach with children [18,42,43]. Serious videogames attempt to change behaviour by integrating theory-driven behaviour change techniques (e.g., goal setting, problem solving) into a fun and engaging videogame [36,42,44] i.e., they attempt to integrate the “serious” aspects of behaviour change with the “fun” aspects of an entertainment-oriented videogame. Advantages of delivering behavior change interventions in a videogame format include high fidelity to intervention design and content; consistent delivery (i.e., delivered in the same way to every participant); and familiarity. Thus, promoting FV intake to children using a serious videogame delivered over the internet would be convenient, familiar, and delivered with high fidelity. This paper presents the outcome evaluation of an online serious videogame promoting FV to children that systematically varied action and coping implementation intentions and examined the short and longer term effects on FV intake.

## Methods

### Study design

The methods have been described in detail elsewhere, but are briefly summarized here [37]. The outcome evaluation used a four group, randomized design with three data collection periods: baseline, post 1 (immediately post-intervention, which was approximately 3 months after baseline), and post 2 (approximately 3 months post-intervention). The child was randomized to condition after both parent and child completed baseline data collection using a random numbers table generated by the study biostatistician.

All groups played the 10-episode online videogame. The groups varied only on type of implementation intention created (Action, Coping, Both, none) after setting a goal to eat FV. Goals were tailored to child’s FV preferences and increased in difficulty to enhance the likelihood of goal attainment [37]. The “Action” group set a FV goal and then created an action plan (i.e., implementation intention) specifying how (i.e., who, what, when) they would meet the goal. They did this by sequentially selecting from a menu of pre-determined categories and choices developed in partnership with children (e.g., “Ask Mom to buy the fruit I need, and put my challenge sheet on my bathroom mirror when I finish playing Squire’s Quest!). Children assigned to the “Coping” group set a goal to eat more FV; they then created a coping plan (i.e., implementation intention) that identified a potential barrier that might keep them from meeting their goal; they did this by selecting from a menu of potential barriers adapted from a previous study with children [36]. They then selected a solution they would try if that barrier was encountered from a menu of barrier-specific solutions (e.g., “If fruit is not always available at home, go grocery shopping with parent/guardian and get the fruit I chose”). Children assigned to the “Both” group set a goal to eat FV, then created both action and coping plans by first creating an action plan, followed by the selection of a barrier and solution using the procedures described above. Children assigned to the “Control “group played the game, but only set a goal to eat FV i.e., they did not create an action or coping implementation intention. As part of goal review which occurred at the beginning of episodes 2–10, children in the Action, Coping, and Both groups reported whether they used the implementation intention they created to meet their FV goal. Episodes were designed to take no more than one hour to complete. Children were asked to attain their FV goal prior to playing the next episode.

### Sample

Inclusionary criteria were: a child in the 4th or 5th grade (~9-11 year olds) who spoke English, had a computer and high speed Internet access, and a parent willing to participate. The primary outcome was child FV intake. An a priori power analysis indicated a sample of 400 parent/child pairs would provide > 80% power at an alpha ≤ 0.05 to detect a small effect size (Cohen’s d = 0.17), allowing for a 30% attrition rate. This effect size translates to detecting a group difference of 0.51 FV servings or more. Recruitment methods included posting flyers in community locations likely to be frequented by parents and children, placing notices in electronic newsletters and on websites, and calling eligible families in the USDA/ARS Children’s Nutrition Research Center volunteer database. Written parental consent and child assent were obtained prior to participation. Human Subject approval was obtained from the Institutional Review Board at Baylor College of Medicine, Houston, TX, USA (H-18488). The trial was registered with Clinicaltrials.gov (NCT01094004).

### Child intervention (videogame)

#### Squire’s Quest! II

*Saving the Kingdom of Fivealot* (SQ2) is a 10-episode, online videogame designed to encourage 9–11 year old children to consume at least 5 servings of FV each day. Examples of serving sizes taught in the videogame were ½ cup sliced or chopped FV or 1 cup leafy greens. SQ2 is an update and enhancement of the original videogame (*Squire’s Quest!)* (SQ!) played on computers in a classroom setting by 4th grade children [18]. The SQ2 design framework included multiple theories to guide various aspects of behavior change: Social Cognitive Theory for personal and environmental factors that influence FV intake [15]; Self Determination Theory for motivation to eat more FV [45]; Behavioral Inoculation Theory for resistance to temptations not to eat FV [46]; Maintenance Theory for long-term behaviour change (i.e., continued FV intake) [47]; and the Elaboration Likelihood Model for enhanced information processing (e.g., attracting and maintaining attention) [48]. The design framework [37] guided both the behavioural and entertainment-oriented components of the game, including behavioural procedures [15,45-47]; content [15]; character looks [48], personalities [48], and actions [15,49]; storyline [48], and dialogue [15,45,48,49]. For a more detailed description, see Thompson et al. [37].

After both parent and child completed baseline data collection, the child became a Squire (i.e., a Knight in training) in the Kingdom of Fivealot. The Squire’s mission was to learn the sacred knowledge and skills (e.g., behavioural components designed to increase FV intake) needed to become a Knight to help King Brockwell and Queen Nutritia save the Kingdom from invaders (i.e., snakes and moles) attempting to overthrow the Kingdom by destroying its bountiful FV crops. Squires (players) were assisted in their effort by a “Knight in training” toolkit (e.g., measuring cups and spoons, an apron with the study logo) shipped to them prior to initiating gameplay) and the behavioral components led by game protagonists (i.e., characters). Behavior change components included FV knowledge enhancement (e.g., serving sizes, “real” FV vs FV “imposters”, kitchen safety); development of key skills needed to increase and maintain FV intake (e.g., goal setting, problem solving, decision making, self-monitoring, resisting temptation, recipe preparation, asking/negotiation), and demonstrations of how to use strategies (e.g., patterns or schemas) to eat at least 5 servings of FV each day (e.g., the Queen’s schema was 1 FV at breakfast, 2 at lunch, 1 at dinner, 1 at snack) [37]. The story, written by a professional writer, was designed to be relevant [48], entertaining [42], and immersive to children [50,51]. It integrated the behaviour change components into the story in a manner that helped advance game-play. The game characters (i.e., protagonists) served as role models [37] who led the knowledge enhancement components of the game, modelled how to perform key skills and behaviors using a coping style [49], and provided performance feedback to the player [15].

Extensive formative research was conducted during development to ensure SQ2 was developmentally appropriate and appealing to children. Alpha and beta testing identified technical issues prior to the outcome evaluation. A pilot study was conducted to test procedures (enrollment, data collection, intervention delivery) and serve as a final beta test of the online game. During development, a 4th grade teacher reviewed intervention components to ensure age appropriateness and provided feedback on needed changes to ensure comprehension and appeal.

### Parent intervention (electronic newsletters, parent-only website)

Parents received 10 electronic newsletters and access to a 10-installment parent-only website. The parent intervention was connected to the child intervention. When the child was given access to the next episode of the video game, parents received access to the next installment of the parent intervention. The newsletters informed parents about lesson content for each episode of the videogame and provided a vocabulary list of words in the corresponding episode that might be unfamiliar to the child (e.g., “tome” for book), tips for how they could help their child meet FV goals (e.g., have their favorite FV available), and suggestions for how to overcome barriers families commonly encounter when attempting to eat more FV (e.g., barrier: family does not have time for breakfast; tip: Mix a serving of dry cereal with a serving of raisins or other dried fruit and place in a plastic bag to eat on-the-go). The parent website provided information on ways to create a healthy home environment (e.g., grocery shopping tips, guidelines for creating a well-stocked kitchen to make it easier to prepare quick, healthy, and affordable meals). The parent intervention did not vary by group; all parents received the same intervention, regardless of child group assignment.

### Procedures

After baseline data were collected and children were randomly assigned to group, child and parent were each given a unique password that routed them to their assigned intervention. When parents and children were eligible for the next session, separate emails with links to their intervention were automatically generated and emailed to them simultaneously. Alerts notified the intervention staff when a component was completed by parents and children. Children set two types of goals each episode: a goal to eat more F and/or V (i.e., FV goal) and to make a child-friendly FV recipe demonstrated in the game (i.e., FV recipe goal). Episodes varied by whether they promoted F, V, or FV. FV goals were connected to the focus of each episode and were distributed as follows: 3 F, 3 V, 1 F or V, and 3 FV goals. FV goals gradually increased in difficulty and excluded 100% juice and high fat and fried V (HFV) (e.g., French fries). Implementation intentions were created for FV goals only.

### Measurement

Parents and children received an email with a link to a secure, password protected website hosting the questionnaires when it was time for data collection. Parents and children completed separate online questionnaires. Parents provided demographic data at baseline and self-reported newsletter and website usage at post 1. Process evaluation data were collected via logs maintained by study staff, and automatically within the game as children navigated it (e.g., logon rate, responses to questions embedded in the game).

The primary dependent variable was child FV intake. Because preferences for F and V vary [29], secondary analyses assessed F and V intake separately to provide a more detailed view of intervention effects on FV intake. At each data collection period, food and beverage intakes were measured via 3 unannounced 24 hour dietary recalls (2 weekday, 1 weekend day) conducted over the phone by trained staff using the Nutrient Data System for Research (NDSR-2009, Nutrition Coordinating Center, University of Minnesota, Minneapolis, MN) [52]. The dietary data collection team was blinded to group assignment. Servings of F and regular V (i.e., non-fat, non-fried) were calculated using the NDSR output. Three days of F and V intake at each data collection period were averaged to improve estimates of dietary intake. A conservative definition of FV intake was used in the analyses, which included F, excluding 100% juice, and V, excluding HFV. The analyses examined only these variables because the intervention attempted to increase F and regular V intake, but not 100% juice, and HFV.

### Statistical analysis

All statistical analyses were conducted using the Statistical Analysis Software (SAS) (version 9.3, SAS Institute Inc., Cary, NC, 2010). The statistician was not blinded to condition since the analyses required that the data be analyzed by group. Numerical (Skewness, Kurtosis, and Kolmogorov-Smirnov D) and graphical methods tested for data normality or any suspected outlier. Because the data were skewed and not normally distributed, the outcome variables were log-transformed for the analyses. Estimates (i.e. adjusted means) presented in the tables were back-transformed to the original scale.

Baseline demographic characteristics and FV intake were examined to identify group differences using chi-square analysis and analysis of variance (ANOVA) for categorical and continuous variables, respectively.

All analyses were conducted only with children who had three dietary recalls at all three data collection periods (i.e., complete data). Intent-to-treat analyses assessed the influence of drop-outs on intervention effects.

A four-level between-group factor and a two-level within factor (post 1 and post 2) repeated measures analyses of covariance (RM ANCOVA) using mixed-effect models (Proc Mixed procedure in SAS) adjusted for baseline outcome values and selected covariates (e.g., child’s gender, race/ethnicity, total energy intake, parent’s age, and household education). The models evaluated change from baseline to post1 or post 2. Because multiple comparisons were conducted, there was a potential for type I error inflation. Therefore, the Bonferroni correction was applied, resulting in a significant p-value of <0.01, which then was used for all analyses to determine statistical significance.

## Results

### Baseline characteristics

Four hundred parent/child pairs (were successfully recruited in approximately 8 months. Baseline characteristics of the study sample indicated children were almost evenly distributed by gender (female, 52.7%) and were of diverse ethnicity (White-36.8%, Hispanic 27.4%, African American 26.4%). Parents were mostly female (96.3%), White (40.3%), married (77.5%), and 40–59 years old (55.3%). Highest level of household education was predominately post-graduate study (36.7%), and average household income was > $61,000 (57.6%) (Table 1).

**Table 1:** **Frequencies (n) and percentages (%) of baseline demographic characteristics for the study sample, stratified by intervention group**

	**Total**	**CONTROL**	**ACTION**	**COPING**	**BOTH**
	**n ( % )**	**n ( % )**	**n ( % )**	**n ( % )**	**n ( % )**
**Sample**	387 (100.00 )	97 ( 25.06 )	98 ( 25.03 )	95 ( 24.55 )	97 ( 25.06 )
**Child gender**					
Male	183 ( 47.29 )	45 ( 24.59 )	48 ( 26.23 )	43 ( 23.50 )	47 ( 25.68 )
Female	204 ( 52.71 )	52 ( 25.49 )	50 ( 24.51 )	52 ( 25.49 )	50 ( 24.51 )
**Child race/ethnicity**					
Hispanic	106 ( 27.39 )	28 ( 26.42 )	27 ( 25.47 )	26 ( 24.53 )	25 ( 23.58 )
African American	102 ( 26.36 )	23 ( 22.55 )	32 ( 31.37 )	25 ( 24.51 )	22 ( 21.57 )
White	141 ( 36.43 )	33 ( 23.40 )	29 ( 20.57 )	37 ( 26.24 )	42 ( 29.79 )
Other	38 ( 9.82 )	13 ( 34.21 )	10 ( 26.32 )	7 ( 18.42 )	8 ( 21.05 )
**Parent gender**					
Male	15 ( 3.75 )	3 ( 20.00 )	3 ( 20.00 )	5 ( 33.33 )	4 ( 26.67 )
Female	385 ( 96.25 )	97 ( 25.19 )	97 ( 25.19 )	95 ( 24.68 )	96 ( 24.94 )
**Parent race/ethnicity**					
Hispanic	102 ( 26.36 )	28 ( 27.45 )	24 ( 23.53 )	25 ( 24.51 )	25 ( 24.51 )
African American	102 ( 26.36 )	22 ( 21.57 )	33 ( 32.35 )	25 ( 24.51 )	22 ( 21.57 )
White	156 ( 40.31 )	35 ( 22.44 )	36 ( 23.08 )	40 ( 25.64 )	45 ( 28.85 )
Other	27 ( 6.98 )	12 ( 44.44 )	5 ( 18.52 )	5 ( 18.52 )	5 ( 18.52 )
**Highest household education**					
Some College/Tech or Less	124 ( 32.04 )	31 ( 25.00 )	38 ( 30.65 )	29 ( 23.39 )	26 ( 20.97 )
College Graduate	121 ( 31.27 )	26 ( 21.49 )	26 ( 21.49 )	30 ( 24.79 )	39 ( 32.23 )
Post Graduate study	142 ( 36.69 )	40 ( 28.17 )	34 ( 23.94 )	36 ( 25.35 )	32 ( 22.54 )
**Average annual household income**					
≤ $61,000	164 ( 42.38 )	43 ( 26.22 )	44 ( 26.83 )	45 ( 27.44 )	32 ( 19.51 )
> $61,000	223 ( 57.62 )	54 ( 24.22 )	54 ( 24.22 )	50 ( 22.42 )	65 ( 29.15 )
**Parent age**					
20-39	173 ( 44.70 )	46 ( 26.59 )	46 ( 26.59 )	40 ( 23.12 )	41 ( 23.70 )
40-59	214 ( 55.30 )	51 ( 23.83 )	52 ( 24.30 )	55 ( 25.70 )	56 ( 26.17 )
**Parent marital status**					
Married	300 ( 77.52 )	81 ( 27.00 )	73 ( 24.33 )	70 ( 23.33 )	76 ( 25.33 )
Not Married	87 ( 22.48 )	16 ( 18.39 )	25 ( 28.74 )	25 ( 28.74 )	21 ( 24.14 )

**Figure 1:**
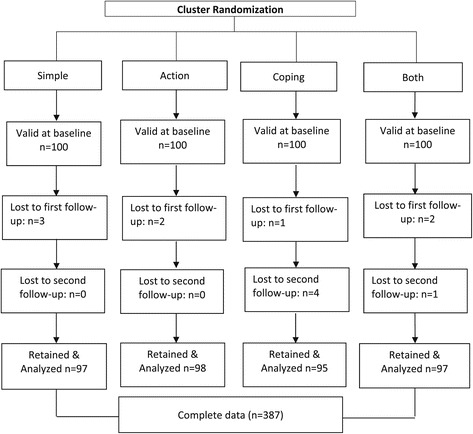
**Consort diagram of the randomization and inclusion process.**

Of the 400 children randomized to condition, 98% had complete data at post 1 (n = 392) and 97% at post 2 (n = 387). Analyses were conducted with children who had complete data at post 2 (n = 387) (Figure 1). There were no group differences in baseline demographic characteristics (e.g., child gender or ethnicity/race, parent age, ethnicity/race, education level) or FV intake (data not shown). There were also no statistically significant differences in demographic characteristics between children included in the analyses (n = 387) and those excluded (n = 13) (data not shown).

### FV intake

At baseline, children consumed an average of 1.8 daily servings of FV, excluding 100% juice and HFV. FV servings were comprised of a mean of 0.5 servings of F and 1.2 servings of regular V (Table 2).

**Table 2:** **Adjusted means (standard error) for the Fruit and Vegetables Consumption (in servings), stratified by group and time (baseline, post 1, and Post 2) using repeated measures ANCOVA** ^**1**^

	**Baseline**	**Post #1**	**Post #2**	**Change** ^**2**^	
	**M(SE)**	**M(SE)**	**M(SE)**	**Post #1**	**Post #2**
**Fruit & vegetable** ^gt **^					
Control	1.86 ( 0.04 )	2.20 ( 0.04 )	1.89 ( 0.04 )	0.35	0.04
Action	1.64 ( 0.04 )	2.37 ( 0.04 )	2.32 ( 0.04 )	0.72***	0.68***
Coping	1.83 ( 0.04 )	2.31 ( 0.04 )	2.11 ( 0.05 )	0.48**	0.28
Both A & C	2.11 ( 0.04 )	2.28 ( 0.04 )	2.05 ( 0.04 )	0.17	−0.06
Total	1.84 ( 0.03 )	2.27 ( 0.03 )	2.07 ( 0.03 )	0.43	0.23
**Fruit**					
Control	0.61 ( 0.04 )	0.84 ( 0.04 )	0.60 ( 0.04 )	0.23	−0.01
Action	0.46 ( 0.04 )	0.82 ( 0.04 )	0.79 ( 0.04 )	0.35	0.33
Coping	0.54 ( 0.04 )	0.84 ( 0.04 )	0.67 ( 0.04 )	0.29	0.13
Both A&C	0.60 ( 0.04 )	0.90 ( 0.04 )	0.69 ( 0.04 )	0.29	0.09
Total ^t***^	0.54 ( 0.03 )	0.82 ( 0.03 )	0.67 ( 0.03 )	0.32**	0.23*
**Regular Vegetables**					
Control	1.18 ( 0.04 )	1.26 ( 0.04 )	1.26 ( 0.04 )	0.08	0.07
Action	1.15 ( 0.04 )	1.47 ( 0.04 )	1.48 ( 0.04 )	0.32	0.33
Coping	1.24 ( 0.04 )	1.37 ( 0.04 )	1.38 ( 0.04 )	0.13	0.14
Both A & C	1.45 ( 0.04 )	1.34 ( 0.04 )	1.32 ( 0.04 )	−0.10	−0.13
Total	1.24 ( 0.03 )	1.36 ( 0.03 )	1.35 ( 0.03 )	0.12	0.11

Abbreviations: M(SE) = adjusted mean ( standard error), “g” = group; “t” = time, “gt” = the interaction of group by time, “ANCOVA” = Analysis of Covariance.

1: Repeated measures ANCOVA was adjusted for gender, race/ethnic group, total energy intake, and parent’s age, education level; adjusted means presented here were back-transformed to original scale (servings).

2: Change from baseline.

Significance level at *p < 0 .01, ** p < 0.001, *** p < 0.0001.

There was a significant group by time interaction for FV intake (F_(6,777)_ = 3.24, p-value < 0.001). At post 1, the Action (p < 0.0001), and Coping (p < 0.001) groups had significant increases in FV intake compared to baseline. However, at post 2, only the Action group maintained these increases (p < 0.0001). The Action group had an almost 50% increase in FV intake at post 1 (0.72 servings), and maintained this increase at follow-up (0.68 servings) (Table 2).

A statistically significant time main effect was observed for F intake (F _(2,780)_ = 28.61, p-value <0.0001). Regardless of group, compared to baseline, F intake increased at both post 1 (p < 0.001) and post 2 (p < 0.01). No significant interaction or main effects were observed for V (Table 2).

### Program participation

Child participation rate was high: 91% of children played all 10 episodes of the videogame. Game-play rates did not vary by group (p = .813, data not shown). Parents’ participation rates varied, however: 33% reported reading 1–3 newsletters; 35% reported reading 4–6 newsletters; and 28% reported reading more than 6 newsletters. Parent self-reported website visitation rates were also highly variable: 55% reported visiting the parent website 1–5 times; 32% 6–10 times; and 28% 11 or more times. Most (94.8%) thought the videogame helped their child eat more FV. When asked to grade the overall program (videogame, newsletters, parent website), most parents gave it an A or B (92.0%) (data not shown).

## Discussion

This study examined longitudinal changes in FV intake after children played a 10 episode theoretically grounded serious videogame that systematically varied implementation intentions. The children who created Action plans as part of goal setting had significantly greater increases in short- and longer-term FV intake.

At baseline, FV intake was substantially below recommended levels for 9–13 year olds [10]. Low FV consumption among children is not surprising and has been observed by others. Fewer than 4% of 9–13 year olds in the US reported consuming 7 or more servings of FV a day in 2006, while <20% consumed at least 5 servings a day [53].

Children in the Action group increased FV intake by .72 servings a day immediately after the intervention. This is somewhat higher than that reported in a review of FV studies with youth and adults which found an average intervention effect of .6 servings a day [13]; it also exceeds effects reported in a recent review and meta-analysis of school-based interventions with children which reported an increase of 0.25 portions a day, excluding juice [54]. The increase at post 1 assessment was less than that observed in the original SQ! game (1.0 servings) [18]. However, the original game included 100% juice and high fat FV when calculating the primary outcome [18]. When examining increases in FV only (.76 servings), immediate post-intervention effects were closer to those observed in the Action group in the current study (.72 servings) [18]. There were also differences in dietary assessment methods and procedures. Although both used 24 hour dietary recalls, the original SQ! used an online dietary recall system that only quantified F, 100% juice, regular V, and high fat V intakes; the original SQ! game also collected 4 recalls on non-consecutive week days, while SQ2 collected recalls on 3 days, consisting of 2 week days and one weekend day.

Although FV intake was still below recommended levels [10], the increase observed in the Action group was nearly 50% above baseline, which is worth noting. To encourage personal mastery [15] (i.e., goal attainment) via self-efficacy, the intervention gradually increased goal intensity rather than starting with a likely more difficult FV goal of at least 5 servings of FV a day [37]. It is possible that a more intensive intervention approach would have resulted in greater increases in FV intake. Future research is needed to investigate this.

Maintenance of intervention effects on FV intake is an important public health issue [12], but mixed effects have been observed [12,13]. Knai et al. [12] examined studies conducted only with youth that had a follow up period of at least 3 months. They found intervention effects ranging from increases of .3 to .99 servings a day. The present study is consistent with those findings. In the current study, the Action group maintained an increase of 0.68 servings FV for 3 months after completion of the intervention, representing a 41% increase over baseline intake. This provides initial evidence that dietary interventions to increase child FV intake long-term would benefit from including Action plans as part of goal setting. A possible explanation of the mechanism through which Action plans might work is that children are not experienced at setting goals to eat more FV [55] and need guidance on how to accomplish this task. Therefore, creating specific plans of how to achieve their FV goals was likely beneficial in that it provided guidance in this area. This is consistent with implementation intention theory [30]. Another possible explanation is that the goals gradually increased in difficulty, and FV options offered during goal setting were tailored to the child’s FV preferences. This may have increased the likelihood the child would strive to meet the goal, particularly when the goal was coupled with a specific plan of how it would be attained. This likely contributed to personal mastery, which is the most effective technique for achieving behavior change [15]. Finally, according to Rothman, satisfaction with behavior change is associated with behavioral maintenance in adults [47]. Satisfaction is a reflection of whether the effort that went into obtaining the goal was “worth it”. It could be that the children who created Action plans perceived meeting goals as easier than children in the other groups (i.e., required less effort) because they had a specific plan of how to accomplish the FV goal, thus making them more satisfied with the outcome. Research is needed to investigate this possibility.

It is somewhat surprising that the group that created both Action and Coping plans did not increase and maintain their FV intake or that the changes were not equal to or similar to those seen in the Action group alone. One possible explanation is that the cognitive effort required to create two types of implementation intentions was too great for children this age. Other possible explanations are that creating both types of plans resulted in a dilution effect or that children this age are not developmentally ready to create and follow two types of plans for one goal. Additional research is needed to investigate these possibilities.

Regardless of group, children increased F consumption at both post 1 and post 2. No treatment effects were observed for V. Although the intervention was not designed to provide a clear explanation for this, it is well established that children prefer F over V [29,56], which may offer a partial explanation. Further, the intervention was powered to detect a small effect of 0.51 serving increase in FV. Increases observed in F and V separately were smaller than this. It is worth noting, however, that the Action group increased and maintained F intake more than 70% over baseline and V intake by nearly 30%. This was not observed in the other groups. Although these increases did not reach the level of statistical significance, the percentage of increase for each is fairly large. Increases of this magnitude are of practical significance and should be investigated in future research.

The majority of children (91%) played all 10 episodes of the videogame. The number of episodes played represents program dose, or the amount of intervention content to which the participants were exposed [57]. Game-play rates did not vary by group, meaning that children in all intervention arms received a comparable program dose. This is higher than that observed in the original game, in which 73.6% of children played all 10 episodes [18]. This could partially be explained by the differences in setting. SQ was played on computers in the school environment following a pre-set schedule, while SQ2 was played online following a schedule determined by the child or parent. This additional flexibility may have increased participation. It could also be partially explained by the high involvement of children in the development of SQ2, which helped ensure the storyline, characters, and game-play features were appealing, that the behavioral components were understood by children, and that technical issues that could impact game-play were identified and addressed prior to the outcome evaluation [37,58].

Although the parents reported they believed the program was beneficial for their child, they did not report high levels of participation in the parent intervention. The low participation rates in the parent intervention were disappointing, but not surprising. Low parent participation rates have been observed in online child-focused programs [19]. Parents are gatekeepers of the home environment [59]; therefore, it is important to identify effective ways to more fully engage parents in online programs attempting to change child behavior.

Strengths of this study include a strong measure of dietary intake; excluding 100% juice and HFV from the primary analyses; a sample powered to detect meaningful change; high participation by children; low attrition rates; a large, multi-ethnic sample; and an intervention that was designed within a multi-theoretical framework with input from the target population. Weaknesses include no group that did not set a goal; age of the children, which may have influenced the accuracy of the dietary recalls; the relatively high income and education levels of the families who participated in the study; and conducting the study in only one geographic region, which makes it difficult to generalize results to other areas. Finally, the intervention utilized a multi-theoretical approach. While this was a strength of the study, it could have potentially diluted the impact or focus of the intervention.

## Conclusions

Serious video games that include complex behavioural procedures appear to be an effective method for increasing FV intake in children. Action intentions were effective at helping children increase and maintain FV intake. Behavior change programs should consider including action plans as part of goal setting to increase both short and longer-term intervention effectiveness.

### Future directions

Future research should include a more detailed analysis of the associations between FV consumption and goal attainment; associations among parent behaviors, the home environment, and child behaviors, including FV intake; and the effect of greater parent involvement on child FV consumption. Even though the socioeconomic level of families participating in this study was fairly high, child FV intake was low. Future research needs to investigate the possible reasons for this.

## Abbreviations

ANOVA: Analysis of Variance
ARS: Agricultural Research Service
e.g.: For example
F: Fruit
FV: Fruit and vegetable
HD: National Institutes of Health, National Institute of Child Health & Human Development
HFV: High fat vegetables
i.e.: In other words
n: Frequency
NDSR: Nutrient Data System for Research
RM ANCOVA: Repeat Measures Analysis of Covariance
SAS: Statistical Analysis Software
SQ!: *Squire’s Quest!*
SQ2: *Squire’s Quest! II: Saving the Kingdom of Fivealot*
USDA: United States Department of Agriculture
V: Vegetable
%: Percent/percentage
